# Resistant starch ameliorated insulin resistant in patients of type 2 diabetes with obesity: a systematic review and meta-analysis

**DOI:** 10.1186/s12944-019-1127-z

**Published:** 2019-11-24

**Authors:** Chenlin Gao, Mingyue Rao, Wei Huang, Qin Wan, Pijun Yan, Yang Long, Man Guo, Youhua Xu, Yong Xu

**Affiliations:** 1 State Key of Laboratory of Quality Research in Chinese Medicine, Faculty of Chinese Medicine, Macau University of Science and Technology, Avenida Wai Long, Taipa, Macau, China; 2 Department of Endocrinology, Affiliated Hospital of Southwest Medical University, Luzhou, 646000 Sichuan China; 3 Luzhou Key Laboratory of Cardiovascular and Metabolic Diseases, Affiliated Hospital of Southwest Medical University, Luzhou, Sichuan People’s Republic of China; 4 Department of Oncology, Affiliated Hospital of Southwest Medical University, Luzhou, 646000 Sichuan China

**Keywords:** Resistant starch, Insulin resistance, Insulin sensitivity, Type 2 diabetes mellitus, Obesity

## Abstract

**Background:**

Resistant starch (RS) is a starch that can be fermented by the microbial flora within gut lumen. Insulin resistance (IR) is a pathophysiological condition related to diabetes and obesity. RS could reduce blood glucose and ameliorate IR in animals, but its effect in human population is controversial.

**Objective:**

The authors conducted a systematic literature review to evaluate the effect of RS diet supplement on ameliorating IR in patients with T2DM and simple obesity.

**Methods:**

Databases that supplemented with RS in ameliorating IR in T2DM and simple obesity were queried for studies on or before August 15, 2018. Parameters including fasting insulin, fasting glucose, body mass index (BMI), homeostatic model assessment (HOMA) etc. were extracted from studies to systemically evaluate effects of RS.

**Results:**

The database search yielded 14 parallel or crossover studies that met the inclusion criteria. The results indicated that there was no significant difference in the amelioration of BMI, HOMA-%S and HOMA-%B in T2DM patients between RS and the non-RS supplementation. However, the fasting blood glucose, fasting insulin and HOMA-IR in T2DM with obesity who supplemented RS were lower than control group, and the subgroup analysis according to the dose of RS supplementation was inconsistency. There was no significant difference between RS and non-RS supplements in patients with simple obesity.

**Conclusion:**

RS supplementation can ameliorate IR in T2DM, especially for the patients of T2DM with obesity, but not in simple obesity.

## Introduction

Type 2 diabetes mellitus (T2DM) is a metabolic disease mainly characterized by chronic hyperglycemia. Latest data from International Diabetes Federation indicate that there are 451 million diabetic patients worldwide in 2017, of which T2DM accounts for about 90% [1]. The pathogenesis of T2DM is not yet clear, and it is generally considered to be a multi-gene related disease that promoted by both genetic and environmental factors [2]. Insulin resistance (IR), is the common characteristic of T2DM [3] and obesity will accelerate its development [4]. Therefore, body control will help to ameliorate IR and improve the efficacy of hypoglycemic medication in T2DM patients [5].

One of the nutritional interventions for metabolic disease is the dietary supplementation of resistant starch (RS), which is a new starch that can’t be digested and absorbed in the small intestine. Studies found RS can be fermented by the microbial flora in the colon. There are mainly 4 types of RS in dietary fiber, namely RS1–4, and RS2 is mostly studied [6, 7]. Compared with other dietary fibers, RS exhibits more advantages against IR [8]. Pre-clinical studies demonstrated that RS2 diets ameliorated IR and increased pancreatic mass in diabetic animals [9, 10]. Similar results were observed in clinical trials that RS supplementation regulated IR, satiety, dyslipidemia and bowel function in T2DM subjects [11, 12].

Although some studies suggest beneficial effect of isolated soluble fiber supplementation on metabolic control in obesity individuals [13], opposite findings were also observed in T2DM or healthy subjects [11, 14–16]. To systematically evaluate effects of RS supplementation on IR in T2DM and simple obese without T2DM, we conducted a systematic review and meta-analysis. The primary outcome within this analysis was HOMA-IR.

## Methods

### Literature search strategy

This systematic review and meta-analysis was conducted in accordance with guidelines set forth by the Preferred Reporting Items for Systematic Reviews and Meta-Analyses. A literature search was performed on PubMed, Embase, Scopus, Web of Science, Cochrane Library, China National Knowledge Infrastructure and ClinicalTrials.gov to obtain articles published or grey articles on or before August 25, 2018. Search terms were “resistant starch” in combination with “insulin resistance”, “insulin sensitivity”, “type 2 diabetes mellitus”, and “obesity”. The search was performed by two authors independently.

### Inclusion criteria

The following inclusion criteria were applied to the articles:

- (1) study design: randomized clinical trial (RCT) that was designed with parallel or crossover manner;
- (2) inclusion cases: individuals with T2DM, and those who were simple obesity but not with T2DM;
- (3) included experiment group was diet with RS supplementation and control group was non-RS supplementation, and outcome measures were quantitative fasting insulin, fasting glucose, BMI and HOMA;
- and (4) was written in English or Chinese.

Two reviewers independently assessed the articles based on the titles and abstracts. Studies that addressed animal or in vitro experiments, lacked original data, not related to RS and IR, redundant publication, case reports, or conference abstracts were excluded. The Cochrane Collaboration’s tool was used to assess the risk of bias according to items including random sequence generation, allocation concealment, blinding of participants and personnel, blinding of outcome assessment, incomplete outcome data, selective reporting, and other bias.

### Definition and data extraction

The RS supplementation was defined as subjects taking RS without other medication. The forms of RS intake by the subjects were either a pure food additive in the daily diet or as a commodity mixture based on RS. The dose of the latter one was converted into pure RS. The control group was generally supplemented with a type of digestible carbohydrate which can’t be fermented. The following information was extracted: study design, subjects, sample size, baseline BMI, treatment and outcomes. An effort was made to email corresponding authors to access information not shown in published articles. All data were independently extracted by CL Gao, MY Rao and W Huang, and confirmed by Q Wan, P Yan, Y Long and M Guo. Disagreements about eligibility and the extracted information were resolved by discussion between all authors, and the corresponding author (Y Xu) ruled on continuing disagreements.

### Statistical analysis

The units of fasting blood glucose and fasting insulin in all the studies were converted into the same, and then data were pooled to calculate the mean difference (MD). The difference of HOMA-IR standard deviation was too large, over 10 folds to other studies, so, these data were pooled to calculate the standardized mean difference (SMD). Random effects model was adopted to facilitate generalizability of results. Statistical heterogeneity was assessed using Q-tests and the I^2^ statistic. All analyses were carried out using Review Manager software, version 5.0 (Cochrane, Copenhagen, Denmark).

## Results

**Fig. 1:**
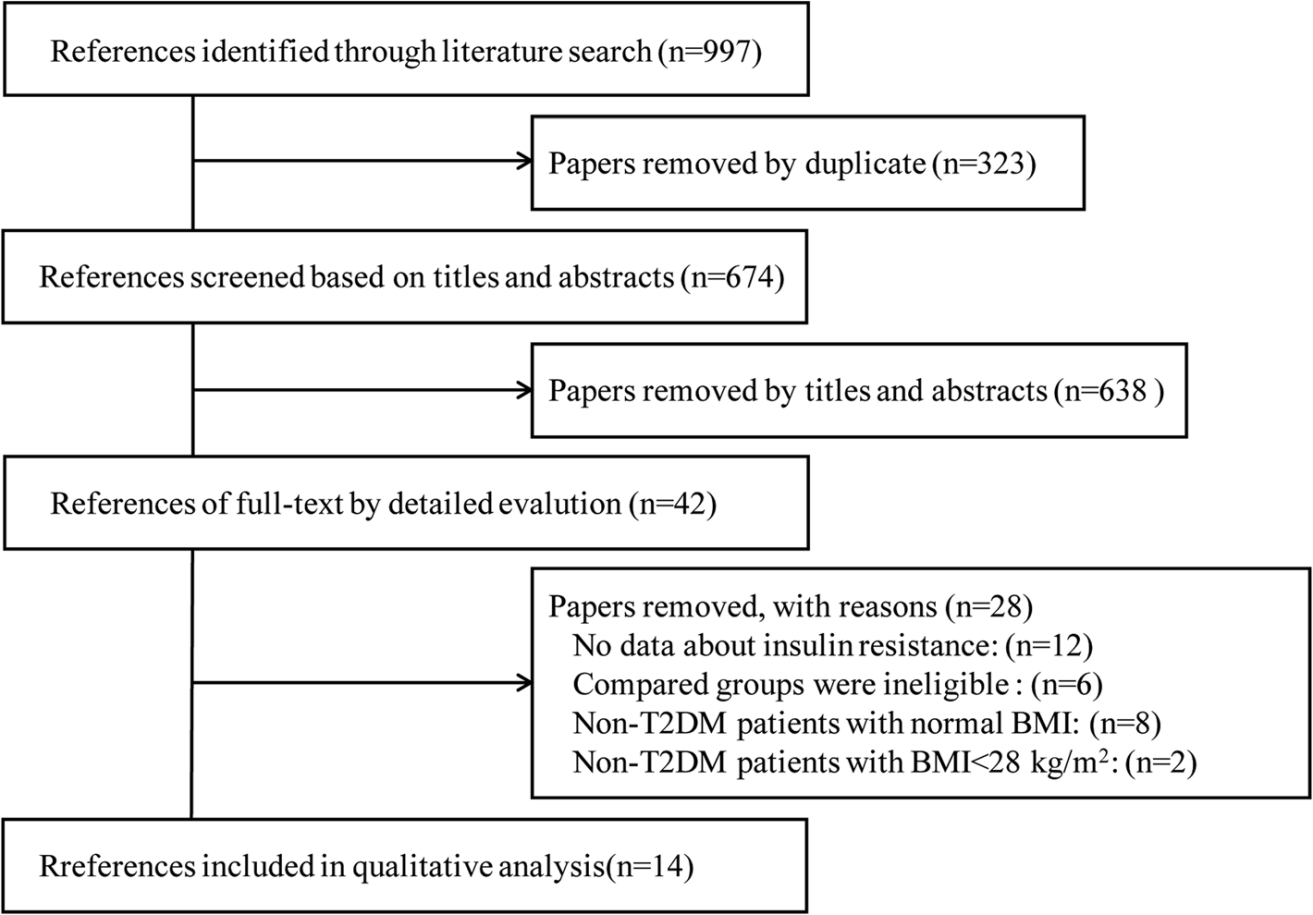
Flow diagram for reference selection

**Fig. 2:**
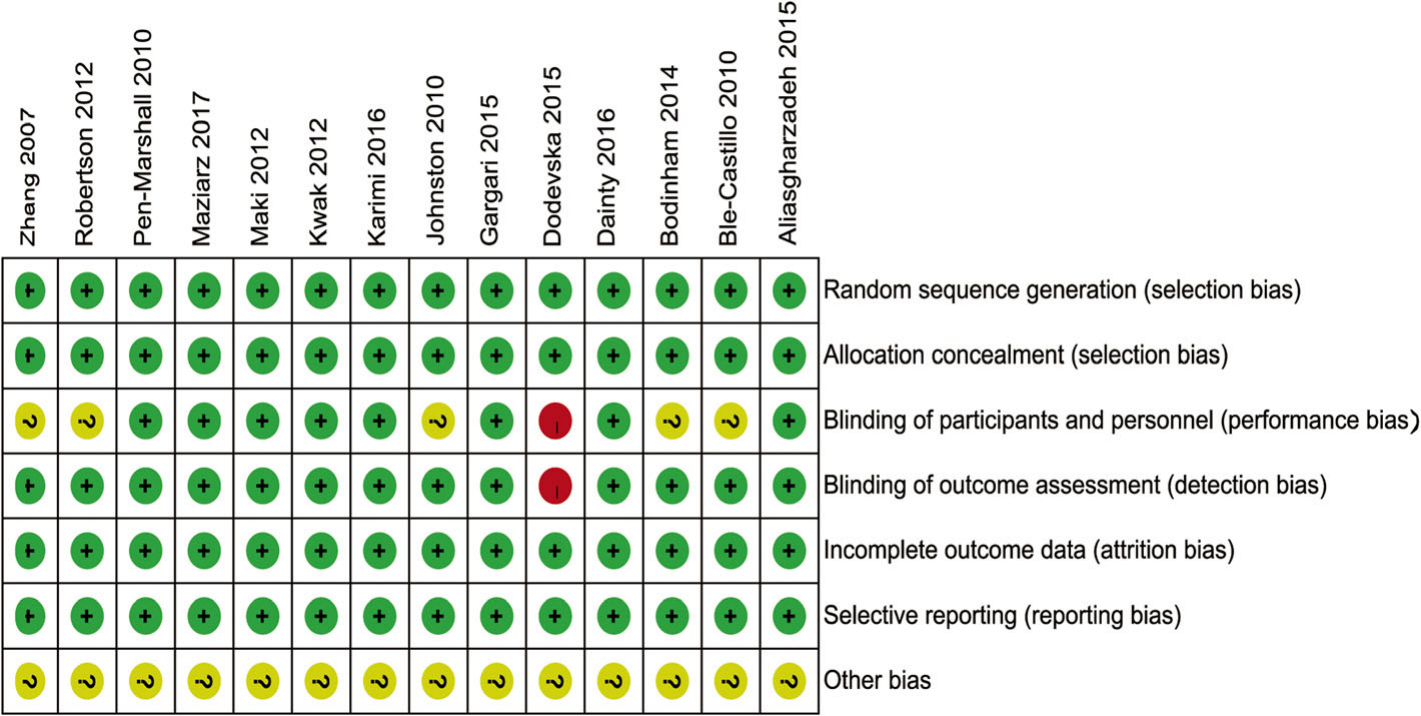
Risk of bias summary for included studies

Our search yielded 997 studies for initial review. After screening titles and abstracts, 42 articles with full-text were reviewed. Twenty-eight articles did not meet inclusion criteria, and the remaining 14 articles comprised with 515 subjects were finally included in the present study [11, 16–28] (Fig. 1). There were 6 studies for simple obesity (without T2DM), and 8 studies for T2DM (6 T2DM with obesity (BMI ≥ 28 kg/m^2^) and 2 T2DM without obesity) (Table 1). Subgroup meta-analysis will be performed based on this feature. According to analysis, the included studies have a lower risk of publication bias (Fig. 2). Only 1 study [28] did not report whether the study was blind or not.

**Table 1:** Study characteristics for the included

Study	Study design	Subjects	Sample Size (n)	Gender (Males/n)	Age (years) RS vs. Control	Baseline BMI (kg/m^2^) RS vs. Control	RS dose (g/d)	Duration (w)	RS total dose (g)	Control	Washout period (w)
Ble-Castillo 2010	R,B, C	T2DM	28	4/28	51.7 ± 5.6	34.89 ± 2.32	8.16	4	228.5	Soy milk	Declared none
Bodinham 2014	R, SB, C	T2DM	17	12/17	55 ± 2.4	30.6 ± 1.3	40	12	3360	RDS	12
Karimi 2016	R, DB, P	T2DM	56	0/56	49.5 ± 8.0 48.6 ± 7.9	31.5 ± 4.5 31 ± 4.9	10	8	560	Maltodextrin	Non existent
Kwak 2012	R, DB, P	T2DM	85	47/85	51.7 ± 2.03 49.4 ± 1.74	25 ± 0.49 24.5 ± 0.37	6.51	4	182.3	Cooked refined rice	Non existent
Zhang 2007	R, SB, C	T2DM	40	17/40	51.1 ± 7.9 52.2 ± 11.1	27.94 ± 2.5 26.87 ± 2.22	30	4	840	Wheatmeal	1
Robertson 2012	R, SB, C	T2DM	15	8/15	48.9 ± 3.9	33.8 ± 1.9	40	8	2240	RDS	8
Aliasgharzadeh 2015	R,DB,P	T2DM	55	0/55	49.2 ± 9.6 49.6 ± 8.4	31.8 ± 4.5 30.8 ± 5.2	10	8	560	Maltodextrin	Non existent
Gargari 2015	R,DB,P	T2DM	60	0/60	49.5 ± 8.0 49.6 ± 8.4	31.5 ± 4.5 30.8 ± 5.2	10	8	560	Maltodextrin	Non existent
Dainty 2016	R, DB, C	Obesity	24	16/24	55.3 ± 1.59	30.2 ± 0.57	25	8	1400	Control wheat bagel	4
Maki 2012	R, DB, C	Obesity	33	11/33	49.5 ± 1.6	30.6 ± 0.5	30	4	840	Control starch	3
Johnston 2010	R, SB, P	Obesity	20	12/20	45.2 ± 3.55 50.1 ± 4.05	31.3 ± 1.70 30.4 ± 1.15	40	12	3360	RDS	Non existent
Penn-Marshall 2010	R, DB, C	Obesity	17	8/17	36.6 ± 1.55	37.7 ± 2.0	12	6	504	Control bread	2
Maziarz 2017	R, DB, P	Obesity	18	3/18	31.0 ± 3.0 31.2 ± 4.2	34.8 ± 1.5 30.6 ± 1.5	30	6	1260	Control muffins	Non existent
Dodevska 2015	R	Obesity	47	19/47	58.4 ± 6.12 57.0 ± 6.13	33.06 ± 5.59 31.07 ± 5.03	8.3	52	3029	Fibre (contain RS)	Non existent

*Abbreviations: T2DM* type 2 diabetes mellitus, *R* randomized, *B* blind, *DB* double-blind, *SB* single-blind, *C* crossover, *P* parallel, *RS* resistant starch, *RDS* rapidly digestible starch, *w* weeks

### BMI

**Fig. 3:**
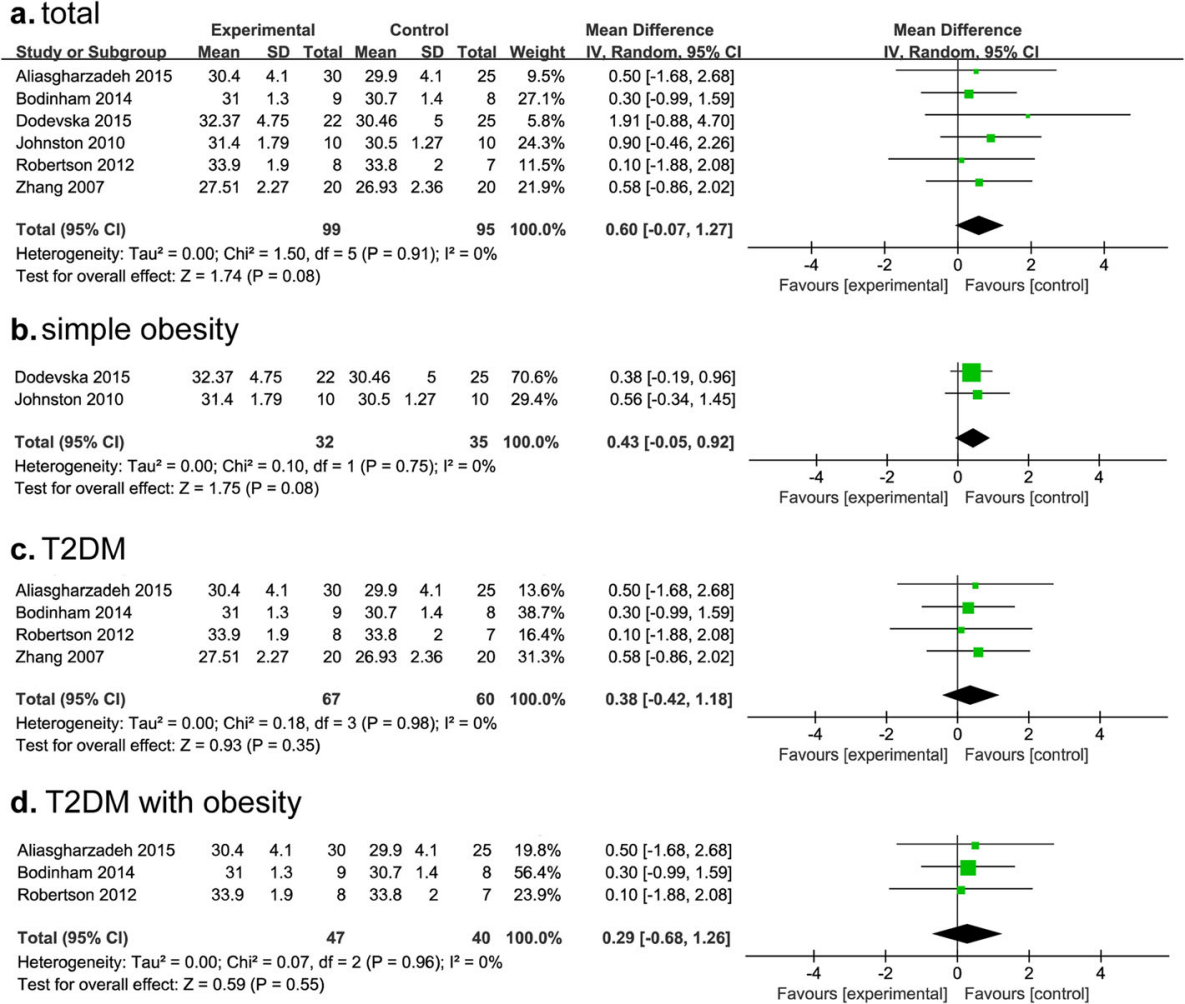
Findings of a meta-analysis of studies with continuation data on improvement in BMI for RS vs control groups, in terms of estimated MD and 95% CIs. (**a**, for whole subjects; **b**, for simple obesity; **c**, for T2DM; **d**, for T2DM with obesity)

BMI in T2DM and obesity patients who were supplemented with or without RS was analyzed. Six articles contained BMI data for individuals with RS were included; among thm, 4 articles were for T2DM and 2 for obesity study. Our results did not show RS supplementation on reducing BMI in whole study population (MD, 0.60; 95% CI, − 0.07-1.27; I^2^ = 0%; *P* = 0.08) (Fig. 3a). Similar results were found in simple obese or T2DM patients (Fig. 3b, c). The distribution of baseline BMI was more dispersed (mean: 24.5–34.9) in studies for T2DM. Subgroup data of T2DM with obesity showed that there was also no significant difference (T2DM with obesity: MD, 0.29; 95% CI, − 0.68-1.26; *P* = 0.55; I^2^ = 0%; Fig. 3d). However, BMI was significantly lowered after RS supplementation than that in the control group in T2DM (*P* < 0.05), although the data can’t be pooled in 2 studies. No statistical difference was found in 1 study for simple obesity (*P* = 0.052) (Additional file 1: Table S1).

### Fasting blood glucose (FBG)

**Fig. 4:**
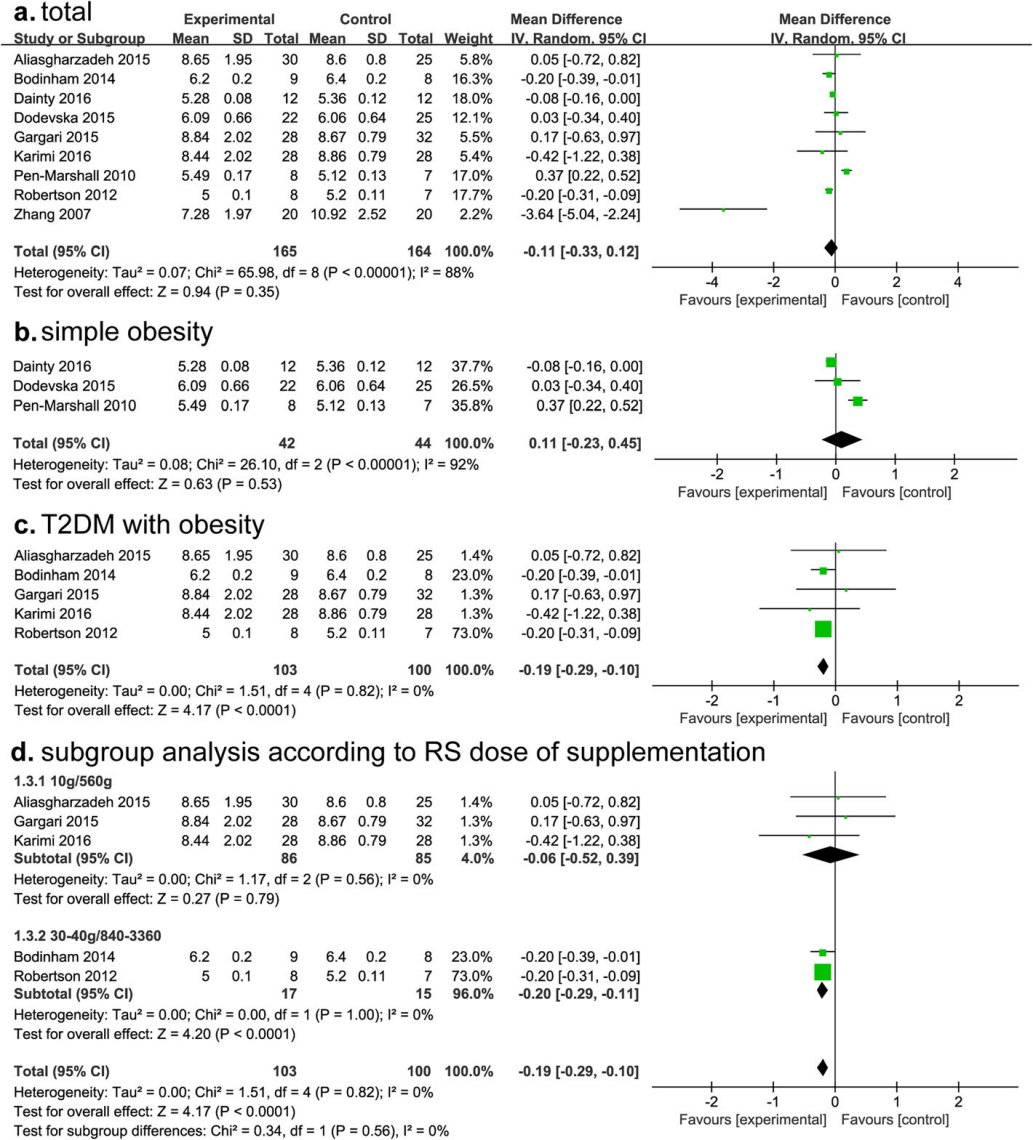
Findings of a meta-analysis of studies with continuation data on improvement in fasting glucose for RS vs control groups, with estimated MD and 95% CIs. (**a**, for total subjects; **b**, for simple obesity; **c**, for T2DM with obesity; **d**, subgroup analysis according to RS dose of supplementation)

A total of 11 studies analyzed fasting blood glucose, including 6 for T2DM and 5 for simple obesity. Although RS consumption did not show statistical significant effects on reducing fasting glucose level in whole cases (*P* = 0.35) (Fig. 4a), subgroup analysis showed that RS supplementation can significantly reduce FBG in T2DM with obesity (MD, − 0.19; 95% CI, − 0.29- -0.10; *P* < 0.0001; I^2^ = 0%), but not in simple obesity population (*P* = 0.53) (Fig. 4b, c).

The weight of Robertson study exceeded other studies too much; we therefore further carried out subgroup analysis according to RS supplemental dose. Interestingly, we found the reduction of FBG in group with RS 30–40 g/day (840–3360 g, total dose) was more significant than that with 10 g/day (560 g, total dose) (MD, − 0.19; 95% CI, − 0.29- -0.1; *P* < 0.0001; I^2^ = 0%) (Fig. 4d).

Studies for simple obesity were too few to carry out subgroup analysis. Data from 6 reports that study the change of FBG after RS intervention showed that FBG was lower than control group in T2DM and obesity patients, but not all the differences were statistically significant (Additional file 1: Table S1).

### Fasting insulin

**Fig. 5:**
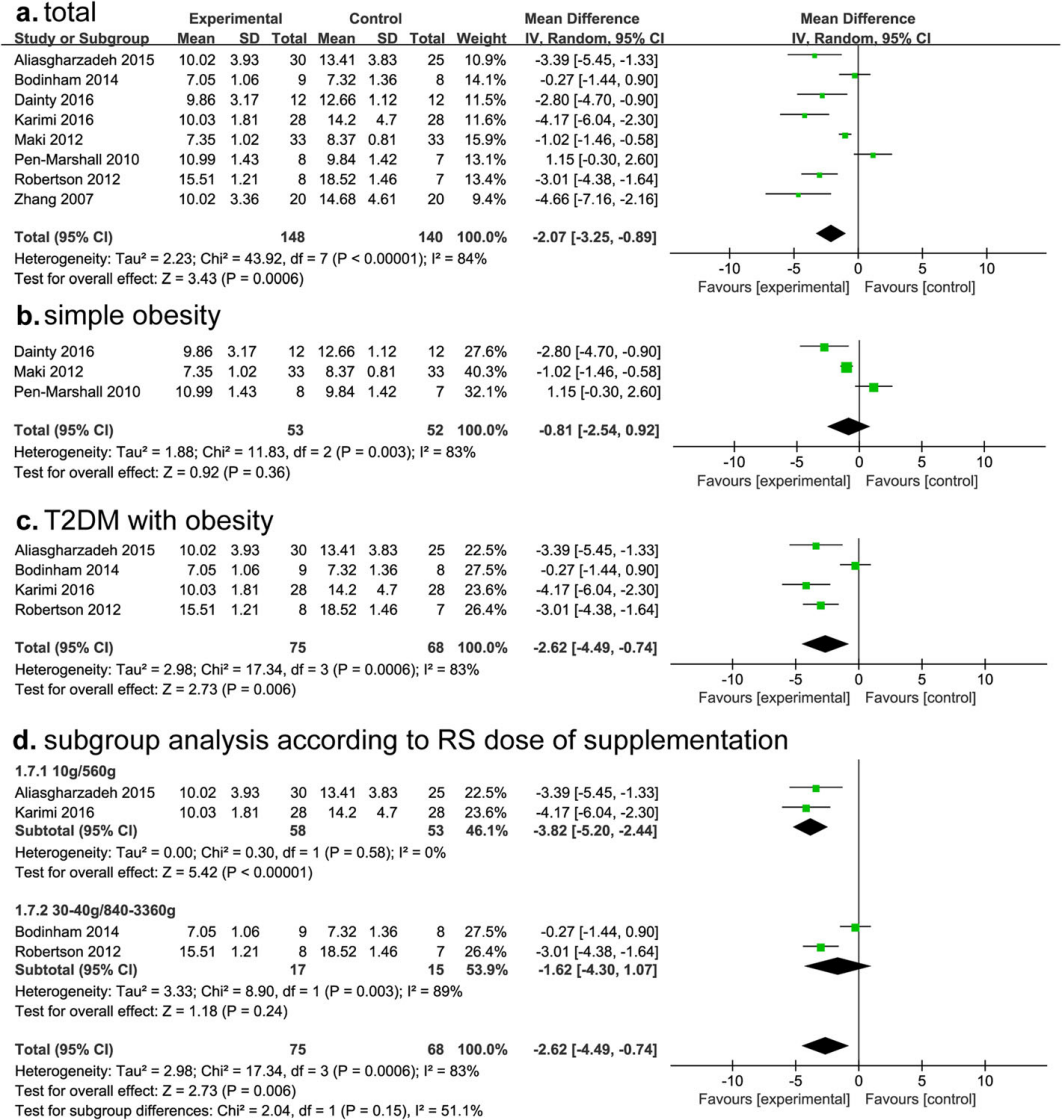
Findings of a meta-analysis of studies with continuation data on improvement in fasting insulin for RS vs control groups, with estimated MD and 95% CIs. (**a**, for total subjects; **b**, for simple obesity; **c**, for T2DM with obesity; **d**, subgroup analysis according to RS dose of supplementation)

A total of 10 studies analyzed fasting insulin for whole subjects, including 5 studies for T2DM and 5 for simple obesity. After RS consumption, fasting insulin levels was lower than control group, but there was significant heterogeneity (I^2^ = 81%) (Fig. 5a). Moreover, the decrease of fasting insulin was not statistically significant in simple obesity, but in T2DM with obesity, and heterogeneity still existed (MD, − 2.92; 95% CI, − 4.49- -0.74; *P* = 0.006; I^2^ = 83%) (Fig. 5b, c).

To eliminate the heterogeneity, we performed subgroup analysis according to RS dose, and found fasting insulin reduction in the group of 10 g/day (560 g, total dose) was more significant than group of 30–40 g/day (840–3360 g, total dose) (MD, − 3.82; 95% CI, − 5.2- -2.44; *P* < 0.00001; I^2^ = 0%) (Fig. 5d). According to the 7 studies investigated correlation between fasting insulin and RS supplementation, although level of fasting insulin was lower than control group in T2DM and obesity, most studies showed with no statistical significance (Additional file 1: Table S1).

### HOMA-%S, HOMA-%B and HOMA-IR

HOMA-%S is an index to evaluate the insulin sensitivity, HOMA-%B is used to evaluate the function of islet β cells, and HOMA-IR is used to evaluate the IR level. We compared these results in T2DM and obesity. We found RS consumption did not ameliorate HOMA-%S in the included 2 studies (MD, 0.51; 95% CI, − 0.43- 1.45; *P* = 0.29; I^2^ = 49%) (Additional file 2: Figure S1a); similar result was observed for that of HOMA-B% in the 4 included studies (2 for T2DM and 2 for obesity) (MD, − 0.14; 95% CI, − 0.71- 0.44; *P* = 0.65; I^2^ = 28%) (Additional file 2: Figure S1b).

**Fig. 6:**
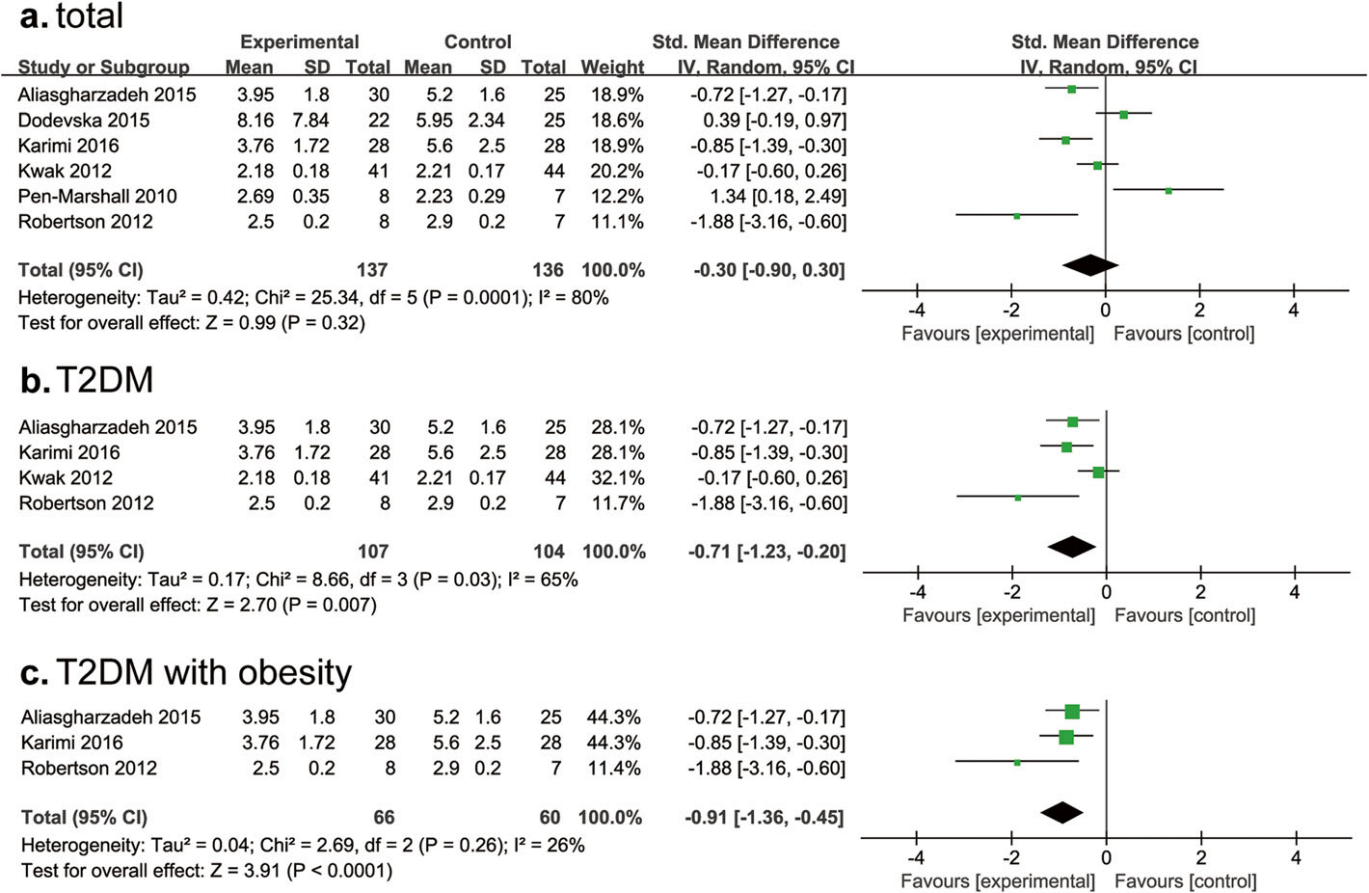
Findings of a meta-analysis of studies with continuation data on improvement in HOMA-IR for RS vs control groups, with estimated SMD and 95% CIs. (**a**, for total group; **b**, for T2DM; **c**, for T2DM with obesity)

As RS consumption did not lower HOMA-IR in whole subjects, and there was significant heterogeneity (I^2^ = 80%) (Fig. 6a), we further conducted subgroup analysis based on BMI (BMI < 28 kg/m^2^, BMI ≥ 28 kg/m^2^). We found HOMA-IR in T2DM after RS consumption was significantly lowered than control group (*P* = 0.007; I^2^ = 65%) (Fig. 6b), and the amelioration of HOMA-IR in T2DM with obesity was statistically significant (MD, − 0.91; 95% CI, − 1.36- 0.45; *P* < 0.0001; I^2^ = 26%) (Fig. 6c). There are five studies studied change of HOMA-IR after RS intervention, with 2 showed positive results and 3 negative results (Additional file 1: Table S1). The measures could not be pooled for meta-analysis due to data type.

## Discussion

Many RCTs concerning the relation between RS and IR have been conducted [16–29], and some systematic reviews about the effects of RS on serum cholesterol and bowel function have been suggested [29, 30], but there exist conflict conclusions among these studies. In the present study, we made a systematic review and meta-analysis involving 14 RCTs of parallel or crossover study; to our knowledge, this was the first systematic analysis to evaluate the role of RS supplementation in ameliorating IR in T2DM and simple obesity.

We found the effect of RS supplementation on IR amelioration in T2DM with obesity was better than T2DM. The blood glucose and IR was ameliorated significantly after RS supplementation, and there was some inconsistent for that of blood insulin. We noted that the dosage of RS supplementation could affect IR amelioration, 30–40 g/day decreased FBG (*P* < 0.0001, I^2^ = 0%) while 10 g/day was enough for that of fasting insulin (*P* < 0.00001, I^2^ = 0%). So, we speculated the inconsistent of blood insulin may be related with the daily/total amount of RS. As subgroup analysis on baseline BMI in T2DM may have an unpredictable bias, multiple clinical trials are necessary to evaluate its weight as an independent factor.

Obesity was a well-recognized high risk factor for T2DM, but this study found RS consumption had no significant effective on IR in simple obesity but in T2DM. RS could not be digested and absorbed in the small intestine, but could be fermented by the microbial flora within the colon [31, 32]. Existing evidence suggests that it can modulate the composition of gut microbial and increase the formation of short chain fatty acids (SCFAs) in the process of intestinal fermentation [33, 34]. SCFAs had been shown to increase insulin sensitivity, improve glucose tolerance, and reduce β-cell apoptosis in obese and diabetic animals [35, 36]. In this sense, effect of RS on IR might be related with the increase of specific intestinal flora.

There were some limitations in this study. First, the amount of studies included in this meta-analysis was small, and some studies have small sample size, so the random error was existed and migration of results may occur. Second, the oral glucose tolerance test (OGTT) is used to assess IR in clinical practice commonly [37], but none of the included studies conducted these tests. Finally, this systematic review included many crossover studies; data from these studies may affect the accuracy of the results because of the elution period, and more prospective clinic trails are necessary to clarify the impact of RS supplementation on the prevention and treatment of metabolic diseases. As we have controlled migration in the process of this meta-analysis, the above limitations did not influence our conclusion that RS supplementation can ameliorate IR in T2DM, especially in patients with obesity.

## Conclusions

This meta-analysis indicates that RS supplementation can ameliorate IR in T2DM, especially in patients with obesity.

## Supplementary information

**Additional file 1: Table S1.** Changes from base line (1st treatment) of endpoints after diet intervention.

**Additional file 2: Figure S1.** Finding of meta-analysis of studies with continuation data on improvement in HOMA-S% and HOMA-B% for RS vs control groups, with estimated SMD and 95% CIs. (a, HOMA-S%; b, HOMA-B%).

## Data Availability

The dataset supporting the conclusions of this article is included within the article.
