# Supplementary material for: Resistant starch ameliorated insulin resistant in patients of type 2 diabetes with obesity: a systematic review and meta-analysis
Source: Lipids Health Dis. 2019 Nov 24;18:205. doi: 10.1186/s12944-019-1127-z (PMC6875042; doi:10.1186/s12944-019-1127-z)
Supplement: Supplementary file 1 — Additional file 1: Table S1. Changes from base line (1st treatment) of endpoints after diet intervention. [file 12944_2019_1127_MOESM1_ESM.doc]

**Table S1.** Changes from base line (1st treatment) of endpoints after diet intervention

| Study | Subjects | Fasting glucose | | Fasting insulin | | BMI (kg/m^2^) | | HOMA-IR | | Data type |
| --- | --- | --- | --- | --- | --- | --- | --- | --- | --- | --- |
|  |  | RS | Control | RS | Control | RS | Control | RS | Control |  |
| Ble-Castillo 2010 | T2DM | -2.0  (-43,39.5)  mg/dL | 1.0  (-15.75, 19.25)  mg/dL | -2.9  (-6.5,-0.8)  U/L | -1.1 (-4.9,0.0)  U/L | -0.59  (-0.85,-0.29) | 0.09  (-0.14, 0.37) | 2.21  (0.59,3.36) | 1.12  (0.30,2.93) | median (25th, 75th) |
|  |  | P=0.42 | | P=0.796 | | P<0.0001 | | P=0.436 | |  |
| Karimi  2016 | T2DM | -18.6  (-33.9 to -6.75)  mg/dL | -3.6  (-5.2 to 3)  mg/dL | -1.48  (1.3 to -3.84)  U/L | 1.6  (-1.7 to 0.7)  U/L | None | None | -1.08  (-0.73, -2.20) | 0.53  (-2.34, 0.62) | MD  95%CI |
|  |  | P<0.05 | | P<0.05 | | None | | P<0.05 | |  |
| Kwak  2012 | T2DM | NG | NG | NG | NG | None | None | -0.30±0.10 | 0.11±0.15 | Mean±SE |
|  |  | P=0.064 | | P=0.035 | | None | | P=0.045 | |  |
| Zhang  2007 | T2DM | -3.21± 2.34  mmol/L | -0.57 ±1.48  mmol/L | -3.39±3.01  U/L | -1.04±1.94  U/L | 0.22±0.51 | 0.01±0.34 | None | None | Mean±SD |
|  |  | P=0.000 | | P=0.000 | | P=0.026 | | None | |  |
| Aliasgharzadeh 2015 | T2DM | -0.65  (-1.1, 0.05)  mmol/L | -0.05  (-0.35, 0.2)  mmol/L | -21.17  (-28.5, -13.54)  pmol/L | 2.08  (0.7, 3.2)  pmol/L | None | None | -1.55  (-2.1, -0.9) | -0.05  (-0.08, 0.2) | MD  95%CI |
|  |  | NG | | NG | | None | | NG | |  |
| Gargari  2015 | T2DM | None | None | -18.6  (-33.9, -6.7)  U/L | -1.7  (-6.5, 3.3)  U/L | None | None | None | None | MD  95%CI |
|  |  | None | | NG | | None | | None | |  |
| Maziarz 2017 | Obesity | -1588±545 | -790±706 | -908±941  Mean AUC | 518±1197  Mean AUC | None | None | None | None | Mean±SEM |
|  |  | P=0.285 | | P=0.425 | | None | | None | |  |
| Dodevska 2015 | Obesity | -3.33  Change% | 1  Change% | 10.57 Change% | -6.89 Change% | -4.2  Change% | -1.96  Change% | 7.37  Change% | 3.88  Change% | - |
|  |  | P=0.224 | | P=0.288 | | P=0.052 | | P=0.491 | |  |
