# Supplementary figures and images for: Resistant starch ameliorated insulin resistant in patients of type 2 diabetes with obesity: a systematic review and meta-analysis
Source: Lipids Health Dis. 2019 Nov 24;18:205. doi: 10.1186/s12944-019-1127-z (PMC6875042; doi:10.1186/s12944-019-1127-z)

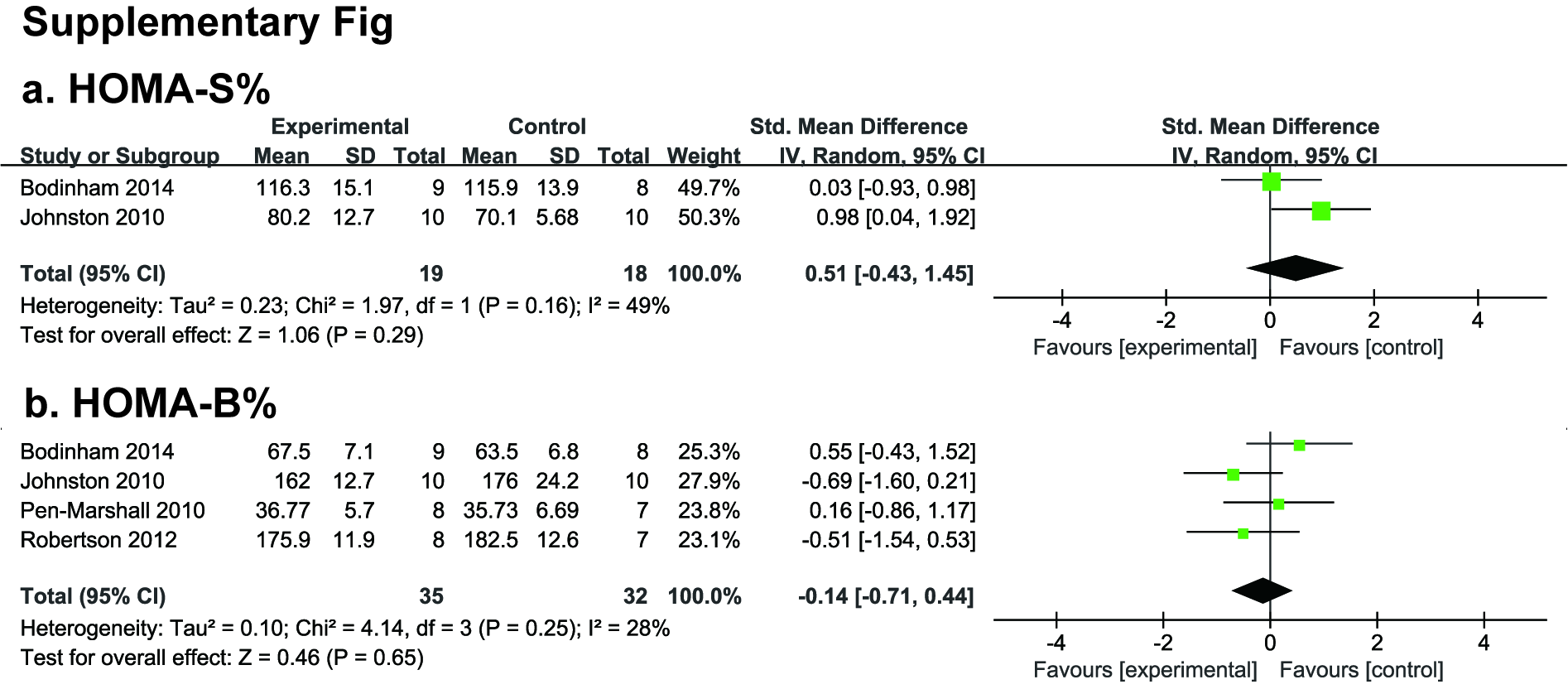

Supplement: Supplementary file 2 — Additional file 2: Figure S1. Finding of meta-analysis of studies with continuation data on improvement in HOMA-S% and HOMA-B% for RS vs control groups, with estimated SMD and 95% CIs. (a, HOMA-S%; b, HOMA-B%). [file 12944_2019_1127_MOESM2_ESM.tif]
